# Immune responses in beta-thalassaemia: heme oxygenase 1 reduces cytokine production and bactericidal activity of human leucocytes

**DOI:** 10.1038/s41598-020-67346-2

**Published:** 2020-06-24

**Authors:** Arnone Nithichanon, Inthira Tussakhon, Waraporn Samer, Chidchamai Kewcharoenwong, Manabu Ato, Gregory J. Bancroft, Ganjana Lertmemongkolchai

**Affiliations:** 1 The Centre for Research and Development of Medical Diagnostic Laboratories, Faculty of Associated Medical Sciences, Khon Kaen University, Khon Kaen, 40002 Thailand; 2 Department of Immunology, National Institute of Infectious Diseases, Tokyo, 162-8640 Japan; 3 Department of Infection Biology, London School of Hygiene and Tropical Medicine, London, WC1E 7HT UK

**Keywords:** Immunology, Microbiology, Biomarkers, Diseases, Health care, Medical research, Molecular medicine, Pathogenesis

## Abstract

Patients with beta-thalassaemia increase the risk of bacterial infections, particularly *Burkholderia pseudomallei* (Bp), the causative agent of melioidosis in Thailand. Impaired immune cell functions may be the cause of this susceptibility, but detailed mechanisms have not been defined. In this study, we observed impaired production of IFN-gamma and IL-10 by whole blood from beta-thalassaemia patients upon stimulation with a range of bacteria-derived stimuli. In contrast, IFN-gamma response via TCR and plasma IgG specific for Bp were still intact. Importantly, mRNA expression of heme oxygenase 1 (HO-1), a potential modulator of immune function, was increased in whole blood from beta-thalassaemia patients, either with or without stimulation with Bp in vitro. Induction of HO-1 by hemin or CoPP in vitro reduced production of IFN-gamma and IL-10 from healthy human PBMCs and decreased bacterial clearance activity of whole blood from healthy controls and beta-thalassaemia, while inhibition of HO-1 by SnPP enhanced both functions in healthy controls. These results were confirmed to some extent in purified human monocytes of healthy controls. Our results suggest a mechanism that excess hemin of beta-thalassaemia patients is a significant cause of immune suppression via HO-1 induction and may underlie the susceptibility of these individuals to severe bacterial infection.

## Introduction

Thalassaemia, a genetic defect in hemoglobin synthesis, is a public health problem worldwide^1^. β-Thalassaemia is a common type of thalassaemia disease which is frequently found in East India, Bangladesh, and Southeast Asia^1^. Bacterial infections were reported as causes of death in thalassaemia patients^2^. In Thailand, thalassaemia and diabetes mellitus are major risk factors for life-threatening infection by *Burkholderia pseudomallei* (Bp), so called melioidosis^3^. In areas where Bp is endemic, most people who have been exposed are seropositive, and develop pre-existing immunity against this bacteria, with only a minority of otherwise immunocompetent individuals progressing to clinical disease^4^.

Understanding melioidosis pathogenesis is crucial to improve prevention of disease, particularly in people with underlying conditions^5^. Recruitment of immune cells including neutrophils, macrophages, natural killer (NK) cells, NK T cells and T cells occurs at sites of Bp infection^6–8^. Bp clearance can be mediated by plasma antibodies which enhance bacterial killing by neutrophils and macrophages^9^. Several pro- and anti-inflammatory cytokines are produced in response to bacterial components which modulate immune homeostasis, resulting in potentially protective inflammatory responses^10^. Interferon-gamma (IFN-γ) has been reported as a crucial pro-inflammatory cytokine to survive melioidosis infection^6,7,11,12^. However, excessive production of pro-inflammatory cytokines can lead to development of tissue damage, organ disfunction, or septic shock^13,14^. IL-10 has been studied in human melioidosis as a potent anti-inflammatory cytokine to counter-balance enhancement of immune functions^15^. Furthermore, a recent study of human plasma cytokine responses in melioidosis revealed the relationship between increasing levels of IFN-γ, IL-6, IL-8, IL-10 and TNF-α to survival of melioidosis patients^16^.

Several impairments of immune response mechanisms are suggested to increase bacterial infection susceptibility in thalassaemia patients^17,18^. For example, alteration of number and function of T cells^19^, B cells^20^ and NK cells^21^, impairment of innate immune functions from neutrophils^22^ and monocytes/macrophages^23^, and reduced activity of complement^24^. Increasing levels of heme due to hemolysis in blood circulation of β-thalassaemia patients has also been suggested as a possible cause of oxidative stress that may lead to infection^25,26^. Heme has detrimental effects on the control of bacterial infections by inhibiting phagocytosis and migration of human and mouse phagocytes^25,26^. Heme oxygenase 1 (HO-1) is an important enzyme for heme catalysis to maintain homeostasis though anti-oxidant and anti-inflammation activities^27,28^. The immunoregulatory actions of HO-1 had been reported to promote Bp infection in mice by increasing serum IL-6, TNF-α and MCP-1, but decreasing IFN-γ production^29^. In mycobacterial infection, HO-1 increased inflammation and bacterial growth in infected mice, and increased bacterial survival in infected human macrophage-like cells^30,31^. These studies strongly suggested that in other circumstances, heme and HO-1 could modulate of host immune responses to increase susceptibility to bacterial infection. However, to date, there is only limited information on the effects of heme and HO-1 in human immune cells taken from patients suffering from thalassaemia.

In this study, we investigated cell mediated immune responses in peripheral blood leucocytes and purified monocytes from β-thalassaemia patients living in the melioidosis endemic region of Thailand.

## Results

### Red blood cell indices from β-thalassaemia patients are decreased compared to non-thalassaemic healthy and diabetes mellitus individuals

Volunteers with no sign of infection were recruited (n = 43) at Nakhon Phanom Hospital. Hematological profile of individuals with β-thalassaemia conditions compared to non-thalassaemic healthy and DM volunteers is shown in Table 1.

**Table 1:** Comparison of demographics, red blood cell (RBC) indices and white blood cell (WBC) parameters between healthy donors without thalassaemia phenotype, β-thalassaemia disease patients (β-thal), and diabetes mellitus individuals (DM).

Parameters	Healthy (n = 20)	β-Thalassaemia (n = 13)	DM (n = 10)	*P* value		
				HC versus DM	HCss versus β-thal	DM versus β-thal
Gender (female:male)	1.5:1	1.6:1	1:1			
Age (year)	32.8 ± 8.4	32 ± 11.2	59.1 ± 7.9	< 0.0001	ns	< 0.0001
RBC indices						
RBC (× 10^3^/µl)	4.8 ± 0.4	3.2 ± 0.9	4.9 ± 0.8	ns	< 0.0001	< 0.0001
Hb (g/dl)	13.5 ± 1.4	6.0 ± 1.7	12.2 ± 1.9	ns	< 0.0001	< 0.0001
HCT (%)	40.4 ± 3.1	19.4 ± 4.2	35.1 ± 5.5	< 0.001	< 0.0001	< 0.0001
MCV (fl)	84.4 ± 4.0	61.8 ± 9.4	73.2 ± 10.8	< 0.05	< 0.0001	ns
MCH (pg)	28.2 ± 1.5	18.0 ± 1.5	25.3 ± 3.8	< 0.0001	< 0.0001	< 0.0001
MCHC (g/dl)	33.2 ± 1.8	29.5 ± 3.4	34.6 ± 1.3	ns	< 0.05	< 0.0001
WBC parameters (× 10^3^/µl)						
Total white blood cells	6.6 ± 1.0	10.5 ± 5.9	8.4 ± 2.7	ns	< 0.05	ns
Neutrophil	3.9 ± 0.6	4.9 ± 2.2	4.9 ± 2.1	ns	ns	ns
Lymphocyte	2.0 ± 0.5	4.5 ± 3.9	1.5 ± 0.4	ns	< 0.05	< 0.0001
Monocyte	0.4 ± 0.3	0.5 ± 0.6	0.8 ± 0.4	< 0.05	ns	< 0.05

Data was shown as mean ± standard deviation.

Statistical significance was determined by using one-way ANOVA with Tukey's multiple comparisons post-test.

*RBC* red blood cell, *Hb* haemoglobin, *HCT* haematocrit, *MCV* mean corpuscular volume, *MCH* mean corpuscular haemoglobin, *MCHC* mean corpuscular haemoglobin concentration, *ns* non-significant.

### Reduction of IFN-γ and IL-10 produced from whole blood samples of β-thalassaemia patients exposed to various bacterial stimuli

β-Thalassaemia disease is thought to be impaired immune response against various type of infections^17^. According to studies on melioidosis, β-thalassaemia and diabetes mellitus are reported as major risk factors for increasing of infection susceptibility^3^. To examine the alteration of immunity in response to Bp, whole blood samples from 13 healthy controls, 15 β-thalassaemia patients and 10 diabetes individuals were stimulated with various bacterial stimuli for 48 h before measured concentration of IFN-γ and IL-10 in supernatant. In this experiment, we addressed on different aspects of immune response by cultured whole blood with medium alone as a background control for cytokine production without stimuli, LPS for activation of innate immune response^32^, PFA fixed Bp for the response against whole bacteria of Bp, and finally Bp-derived FlgK protein for the stimulation through protein processing pathway. To rule out the possibility that uninfected HbE trait individuals may have some impairment on cytokine production upon stimulation, we compared the cytokine results from uninfected HbE trait individuals with healthy without thalassaemic phenotype; IFN-γ and IL-10 production was unimpaired in response to various microbial stimuli in whole blood samples (Fig. S1).

**Figure 1:**
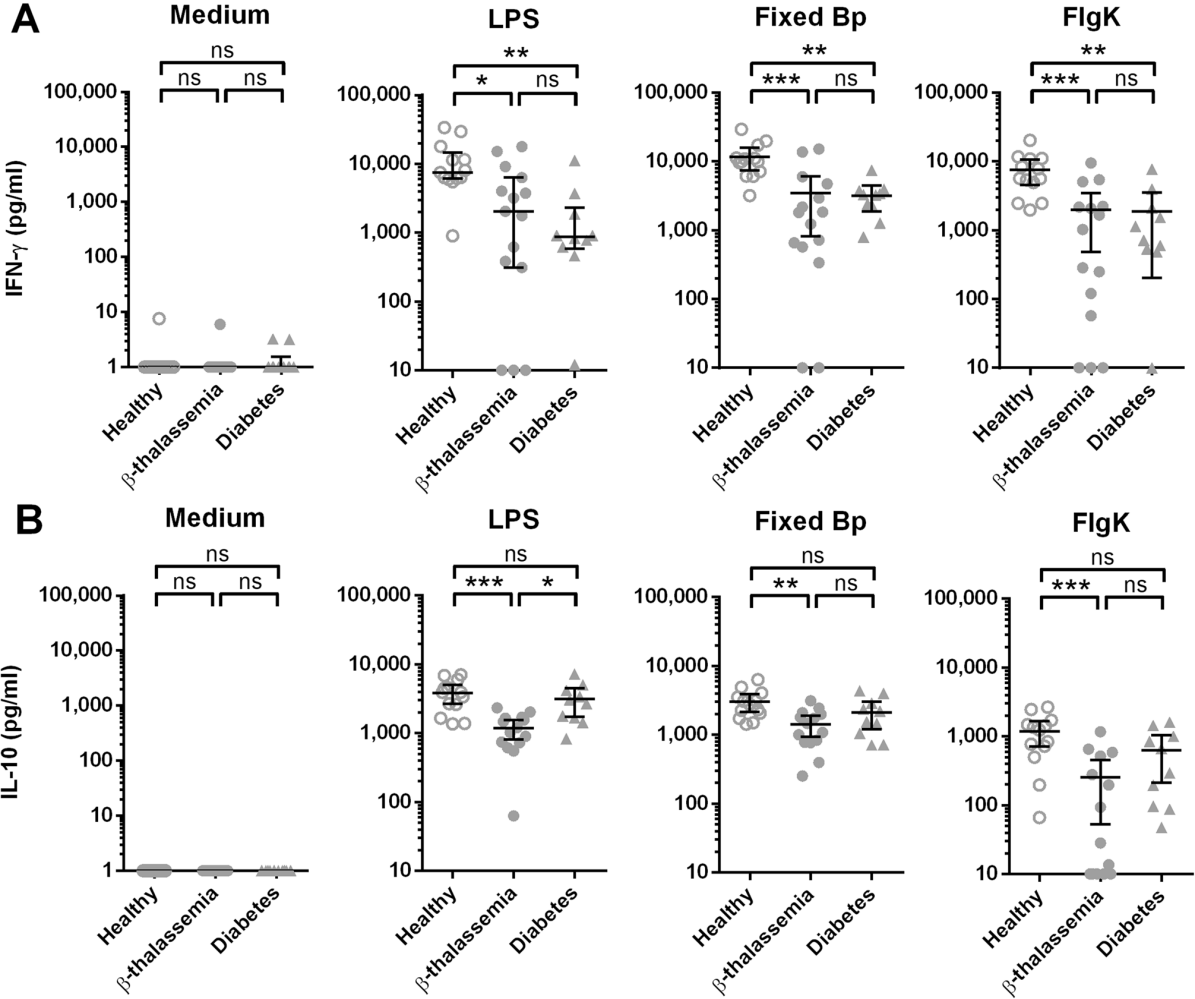
Reduction of IFN-γ and IL-10 produced from whole blood samples of β-thalassaemia patients upon stimulation. Whole blood samples (adjusted the number of lymphocyte plus monocyte at 1.8 × 10^5^ cells) from healthy controls (open circle, n = 13), β-thalassaemia patients (filled circle, n = 15) and diabetes individuals (filled triangle, n = 10) were cultured with medium alone, 10 μg/ml of LPS, 5.4 × 10^6^ CFUs PFA fixed Bp or 10 μg/ml FlgK protein for 48 h. IFN-γ (**A**) and IL-10 (**B**) production upon stimulation in supernatant were measured by ELISA, and the results were shown as scattered dot plot and line at mean with 95% confidence interval. Statistically significant was analyzed by using one-way ANOVA with Tukey's multiple comparisons post-test; *P < 0.05, **P < 0.01, ***P < 0.001, and *ns* non-significant.

IFN-γ production from β-thalassaemia patients after stimulation with LPS, PFA fixed Bp and FlgK are significantly lower than healthy controls (Fig. 1A). In addition, IFN-γ production from individuals with DM is also lower than healthy controls when stimulated with LPS, fixed Bp and FlgK (Fig. 1A). Reduced levels of IL-10 production from β-thalassaemia patients in response to LPS, fixed Bp and FlgK were seen when compared to healthy controls, but the comparisons between healthy controls versus diabetic individuals do not show a significant difference (Fig. 1B). Furthermore, we classified the patients phenotypically into transfusion dependent thalassaemia (TDT) and non-transfusion dependent thalassaemia (NTDT) based on their clinical severity and transfusion requirement^33^ and compared their disease progression and immune (Table S1). Our data in the Table S1 show that 57% of patients with TDT presented splenectomy which was only 22% in NTDT. Moreover, increased ferritin level was demonstrated significantly in TDT compared with NTDT. These results are consistent with a previous report showing severe iron overload that starts early in TDT^34^. In addition, no difference of IFN-γ and IL-10 production was observed between TDT and NTDT patients or between splenectomized and non-splenectomized patients (Fig. S2).

### T cell response though T cell receptor (TCR) and plasma Bp-binding IgG antibody of β-thalassaemia patients are not reduced compared to healthy controls

**Figure 2:**
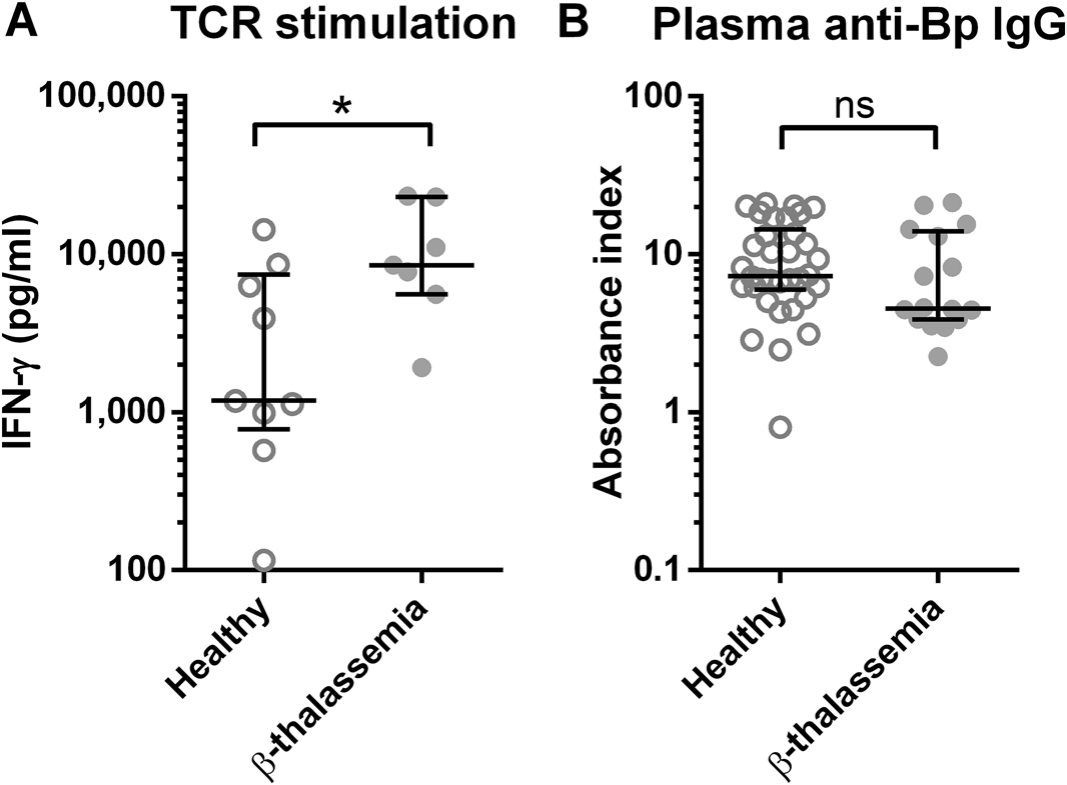
Comparison of plasma anti-Bp IgG antibody level and IFN-γ production upon TCR stimulation between samples from healthy donors and β-thalassaemia patients. Whole blood from healthy control (open circle, n = 9) and β-thalassaemia patients (filled circle, n = 7) were stimulated with anti-CD3 and anti-CD28 antibodies for 48 h before measured for IFN-γ in supernatant by ELISA (**A**). Plasma anti-Bp IgG from healthy controls (open circle, n = 34) and β-thalassaemia patients (filled circle, n = 16) were measured by ELISA (**B**). The results were shown as scattered dot plot and line at median with interquartile range. Statistical analysis was done by using Mann Whitney test; *P < 0.05, *ns* non-significant.

We next sought to examine whether responses from T and B lymphocytes of β-thalassaemia patients are similar or different from healthy controls. Whole blood from 9 healthy controls and 7 β-thalassaemia patients were stimulated with TCR antagonists (anti-CD3 and anti-CD28 antibodies) for 48 h and IFN-γ production in the culture supernatant was measured by ELISA. The results revealed a higher IFN-γ production from β-thalassaemia patients compared to healthy controls (Fig. 2A). Meanwhile, plasma samples from 34 healthy controls and 16 β-thalassaemia patients were measured for Bp-binding IgG antibodies by indirect ELISA. There was no significant difference between Bp-binding IgG antibody levels from healthy controls and β-thalassaemia patients (Fig. 2B). Taken together, this suggests that IFN-γ response via TCR and plasma IgG level from β-thalassaemia patients are intact as healthy controls. Therefore, the causes of alteration in reduction of cytokine production are more likely to be an effect on the other immune functions rather than the adaptive immune responses.

### β-Thalassaemia patients have increased expression of *HO-1* in whole blood culture with Bp

**Figure 3:**
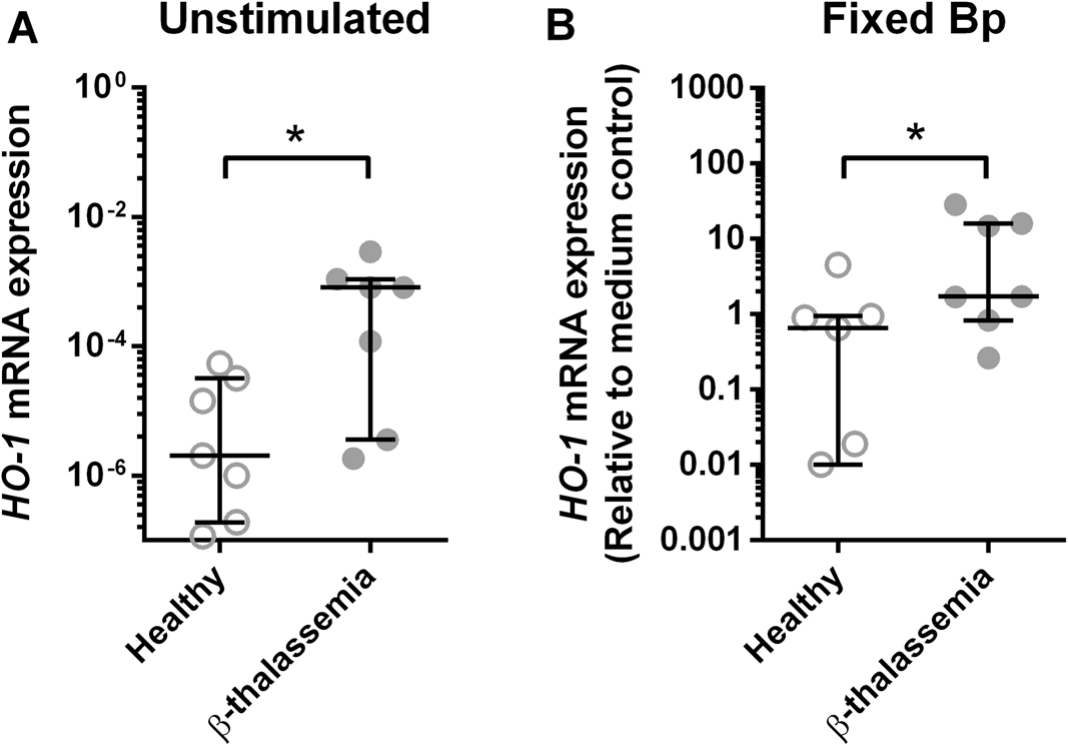
Increasing of whole blood HO-1 expression from β-thalassaemia patients compared to healthy donors with and without fixed Bp stimulation**.** *HO-1* expression from whole blood of healthy controls (open circle, n = 7) and β-thalassaemia patients (filled circle, n = 7) were analyzed by real-time PCR using *GADPH* as internal reference gene. Unstimulated whole blood of healthy and β-thalassaemia were analyzed for ΔCt between *HO-1* and *GADPH* before calculated for *HO-1* mRNA expression by 2^−ΔCt^. PFA fixed Bp stimulated whole blood of healthy and β-thalassaemia were analyzed for ΔCt between *HO-1* and *GADPH*, then ΔΔCt comparing to medium control before calculated for *HO-1* mRNA expression by 2^−ΔΔCt^. Results were plotted as scattered dot plot and line at median with interquartile range. Statistical analysis was done by using Wilcoxon matched pairs signed rank test or Mann Whitney test; *P < 0.05 and **P < 0.01.

β-thalassaemia disease is a condition of impairment of β-globin chain synthesis resulting in increased unpaired α-globin chains. The accumulation of excessed α-globin chains is bound to heme which is finally degraded by heme oxygenases (HO), particularly *HO-1*. To test whether *HO-1* expression was altered, blood samples from 7 β-thalassaemia patients and 7 healthy donors were collected for measuring expression of *HO-1* mRNA by real-time PCR either with or without in vitro stimulation with PFA fixed Bp. The baseline results reveal that *HO-1* expression levels of unstimulated whole blood from β-thalassaemia patients was higher than those of healthy donors (Fig. 3). After Bp stimulation, *HO-1* expression was significantly enhanced in all healthy donors and β-thalassaemia patients. Furthermore, in response to stimulation with fixed Bp, whole blood of β-thalassaemia patients showed significantly increased levels of *HO-1* expression compared with healthy controls (*P* < 0.05). These data indicate that β-thalassaemia patients present the greater up-regulation of *HO-1* expression against Bp when compared with healthy controls.

### Presence of hemin and induction of HO-1 decreased IFN-γ and IL-10 production in response to Bp

**Figure 4:**
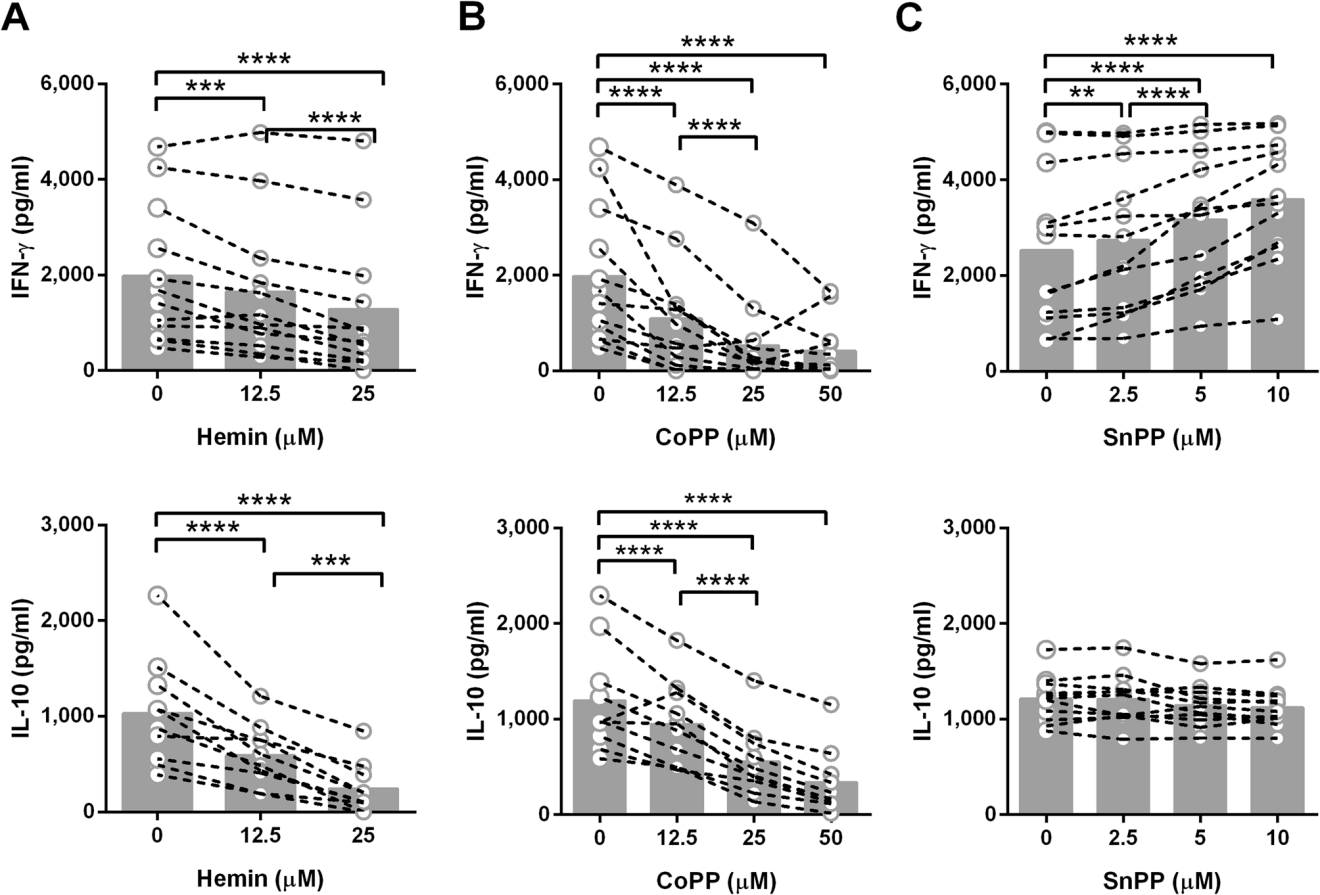
Effect of HO-1 expression associated to IFN-γ and IL-10 production from PBMC after stimulation with fixed Bp in vitro. PBMCs from healthy donors (n = 12) were pre-treated with medium control (0 μM), hemin; HO-1 inducer (12.5 or 25 μM) (**A**), CoPP; HO-1 inducer (12.5, 25 or 50 μM) (**B**), or SnPP; HO-1 inhibitor (2.5, 5 or 10 μM) (**C**) before cultured with PFA fixed Bp for 48 h. Concentrations of IFN-γ (n = 12) or IL-10 (n = 10) in supernatants were measured by ELISA. The data are presented with individual dot plot joined with dash line for sample from the same donor, bar graphs are plotted at median. Statistical analysis was done by using two-way ANOVA with Dunnett's multiple comparisons test; ns, non-significant, **P < 0.01, ***P < 0.001, ****P < 0.0001.

To address the hypothesis that HO-1 is responsible for alteration of immune responses to Bp, PBMCs were isolated from 12 healthy donors and HO-1 mRNA expression after pretreatment with chemical HO-1 inducer or inhibitor was determined by real-time PCR. Hemin (ferriprotoporphyrin IX), a derivative chemical from heme, was used as a representative of heme-induced HO-1 expression^35,36^. Application of cobalt protoporphyrin IX (CoPP) as HO-1 inducer and tin protoporphyrin IX (SnPP) as HO-1 inhibitor are widely used in previous researches^29,30,37,38^. Hemin and CoPP induced *HO-1* mRNA expression as expected (Fig. S3A, B), while SnPP inhibited *HO-1* mRNA expression (Fig. S3C). When cells were pretreated with vehicle control or each HO-1 modulator at various concentrations, reduction of IFN-γ and IL-10 responses were found from both HO-1 inducer, hemin and CoPP, pretreated PBMC in a dose dependent manner (Fig. 4A, B). In contrast, HO-1 inhibitor (SnPP) treatment significantly increased IFN-γ production upon stimulation in a dose dependent manner but did not alter IL-10 production (Fig. 4C). These results support our hypothesis that increasing levels of heme in the blood of β-thalassaemia patients leads to a reduction of IFN-γ and IL-10 production in response to infection or stimulation with various types of bacterial stimuli.

### Presence of hemin and induction of HO-1 expression resulted in the reduced bacterial killing in vitro

**Figure 5:**
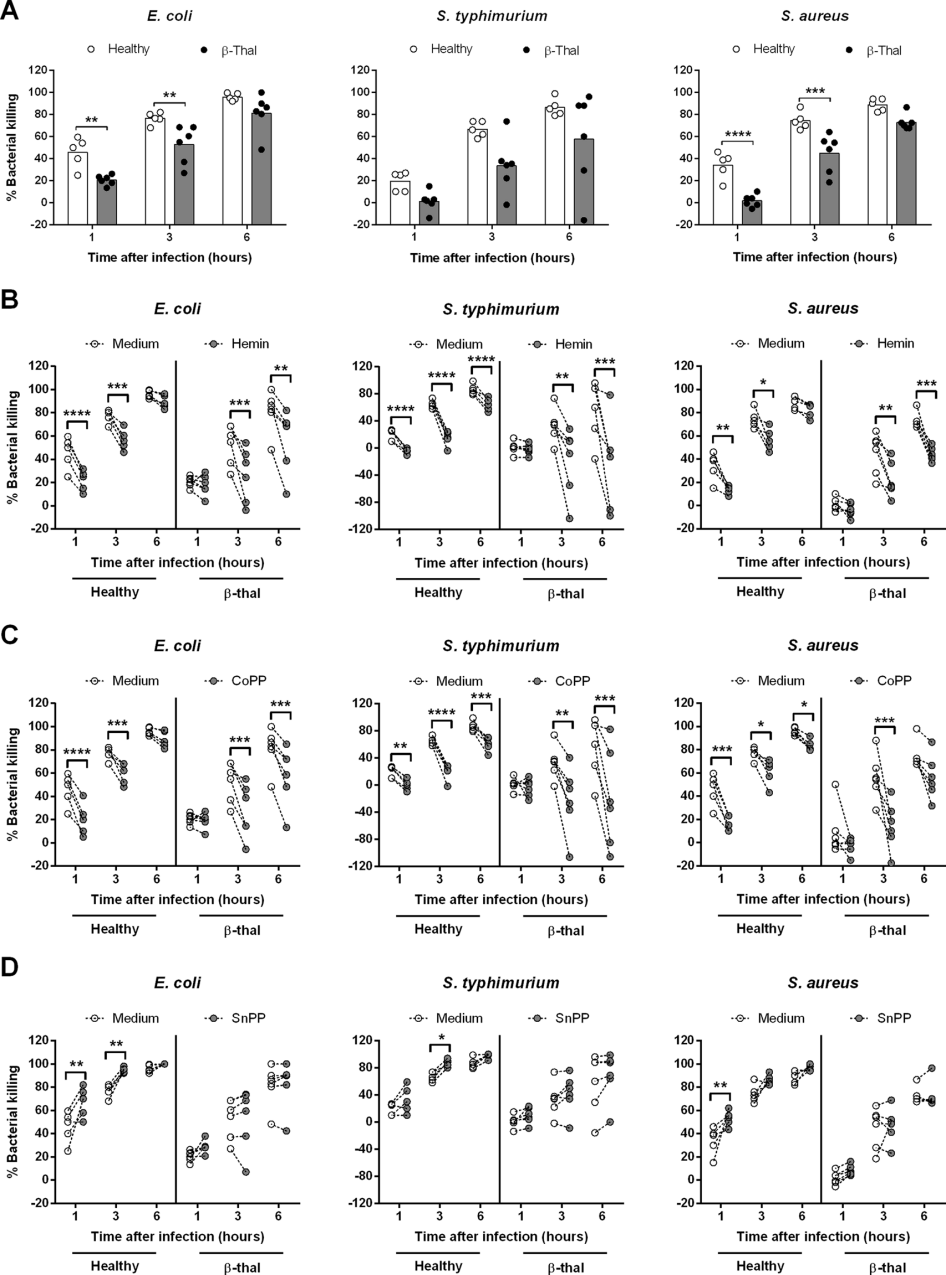
Bacterial killing efficacy of human whole blood from healthy controls and β-thalassaemia patients are related to HO-1 expression. Whole blood from healthy donors (n = 5) or β-thalassaemia (β-thal; n = 6) were pre-treated for 3 h with medium control, hemin at 25 μM, CoPP at 50 μM or SnPP at 10 μM before infection with 10^5^ CFUs of *E. coli*, *S. typhimurium* or *S. aureus* for 1, 3 and 6 h. Total numbers of viable bacteria after infection at various time points were assessed by colony count and calculated as % bacterial killing = ((inoculum bacteria – remaining bacteria)/inoculum bacteria) × 100. Data of % bacterial killing of medium control between healthy versus β-thal is shown as individual dot plots with bar at mean (**A**). Data of % bacterial killing with and without pre-treatment by hemin (**B**), CoPP (**C**), or SnPP (**D**) is shown as individual dot plots with connecting lines of the same donors. Statistical analysis was tested by two-way ANOVA with Bonferroni's multiple comparisons test; *P < 0.05, **P < 0.01, ***P < 0.001, ****P < 0.0001.

Next, we simulated the situation of immune responses occurring in the presence of heme or HO-1 inducer or inhibitor. We addressed the effect of the heme and HO-1 expression on the clearance and cytokines production against common causes of bacterial infection in Northeast of Thailand; *B. pseudomallei*, *Escherichia coli*, *S. typhimurium* and *Staphylococcus aureus*^2^. Whole blood samples of healthy donors or β-thalassaemia patients were pre-treated with medium, hemin, HO-1 inducer (CoPP) or HO-1 inhibitor (SnPP) before being infected with live bacteria and the numbers of surviving bacteria were counted at different time points. In the medium control, bacterial killing efficacy of whole blood from healthy control was significantly more effective than β-thalassaemia patients (Fig. 5A). Induction of HO-1 expression by hemin and CoPP significantly reduced the efficacy of bacterial killing in all bacterial infected whole blood, either from healthy donors or β-thalassaemia patients, regardless of the bacteria species used (Fig. 5B, C). Inhibition of HO-1 expression by SnPP significantly enhanced bacterial killing in whole blood from healthy controls and only slightly increased in β-thalassaemia patients (Fig. 5D). Of note, the delay of bacterial killing activity of whole blood from β-thalassaemia patients was observed, when compared to healthy controls. Meanwhile, bacterial infected whole blood of healthy donors showed significantly induced IFN-γ after treatment with SnPP for 24 h but most of β-thalassaemia patients failed to produce IFN-γ (Fig. S4).

**Figure 6:**
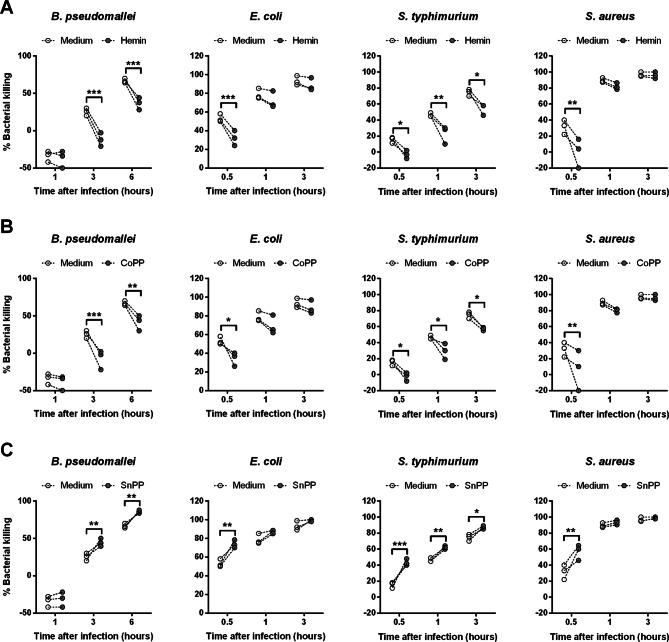
Bacterial killing efficacies of human monocyte are related to inhibition or induction of HO-1 expression. Monocyte from healthy donors (n = 3) were pre-treated 3 h with medium, hemin at 25 μM (**A**), CoPP at 50 μM (**B**) or SnPP at 10 μM (**C**) before infection with 5 × 10^5^ CFUs live Bp for 1, 3 and 6 h or infection with 5 × 10^5^ CFUs of live *E. coli*, *S. typhimurium* or *S. aureus* for 0.5, 1 and 3 h. Total numbers of viable bacteria after infection at various time points were assessed by colony count, calculated as % bacterial killing = ((inoculum bacteria − remaining bacteria)/inoculum bacteria) × 100. Data is shown as individual dot plots with connecting lines of the same donors. Statistical analysis was done by using two-way ANOVA with Bonferroni's multiple comparisons test; *P < 0.05, **P < 0.01, ***P < 0.001.

We then addressed the effect of HO-1 on bacterial killing by using purified human monocytes from healthy controls. The bacterial killing activities at 3 and 6 h post infection with live Bp plus hemin or CoPP pre-treatment were significantly lower than medium control (Fig. 6A, B), whereas SnPP pre-treated monocytes showed significantly higher killing activities (Fig. 6C). Similarly, infection with other types of bacteria also found similar results, suggesting that expression of HO-1 impairs the efficacy of bacterial killing, in both human whole blood and purified monocytes.

## Discussion

β-Thalassaemia is a chronic disease with chronic anaemia caused by mutation in β-globin gene consequently reduced or absent of β-globin chain synthesis resulting to ineffective functions of red blood cells^39^. We observed anaemia in β-thalassaemia patients and DM individuals in this study, especially in β-thalassaemia patients that presented with more severe anaemia. This is consistent with reports that β-thalassaemia is a dyserythropoiesis leading to anaemia^40^, while occurrence of anaemia in DM results from impairment of erythropoietin by the peritubular fibroblasts due to chronic hyperglycemia^41^. Increasing absolute number of total white blood cells and lymphocytes were increased in β-thalassaemia, while DM shown increasing number of monocytes only. These observations are similar as previous reports^42^.

Besides DM, thalassaemia disease is one of the important risk factors of melioidosis^3^. In this study, the whole blood response to *B. pseudomallei* infection of both DM individuals and β-thalassaemia patients showed defects in IFN-γ production. These results in DM are consistent with previously established studies by presenting the reduction of IFN-γ of whole blood from DM against *B. pseudomallei *in vitro^15^ and in PBMC of acute melioidosis patients with DM as underlying condition^43^. These data suggest that both DM individuals and β-thalassaemia patients may have similarly impair T cell immunity in response to *B. pseudomallei* infection. In contrast, we found that β-thalassaemia also impaired IL-10 production when compared with heathy controls, which is not the case in DM^15^. Some beta-thalassaemia/HbE patients have increased plasma IFN-γ level which possibly imply low-grade inflammation that may be caused by an additional independent factor in determining the severity of the anaemia^44,45^. Low-grade inflammation is associated with the reduction of immune functions and proinflammatory cytokines are increasingly found in chronic diseases including type 2 diabetes mellitus (T2DM)^15,45^. Although the causes of low-grade inflammation in T2DM might differ from thalassaemia patients, the consequence of this phenomenon might be similar. Likewise, we found that restimulation of whole blood from beta-thalassaemia/HbE patients with probable low-grade inflammation presented reduced IFN-γ and IL-10 production and bacterial killing activity which may lead to increased susceptibility to melioidosis. Taking these data together, it may imply that the precise nature of the defects in immune response of β-thalassaemia patients against *B. pseudomallei* infection differs from DM individuals and further studies are still needed for investigating these mechanisms.

Next, we studied cytokine responses upon stimulation with various type of stimuli including; LPS, a component of Gram-negative bacterial cell wall, which activates immune response via TLR4^46^, whole intact bacteria of PFA fixed Bp that activate cytokine production via pathways of innate and adaptive immune responses, finally FlgK protein (BPSL0280), a flagella hook-associated protein that can activate IFN-γ production from T cells^43^. We found a clear reduction of IFN-γ and IL-10 secretion from whole blood of β-thalassaemia patients incubated with LPS, fixed whole intact bacteria of Bp, and FlgK protein compared to healthy controls. Other reports in β-thalassaemia found impairments in innate immune functions of NK cells^21^, neutrophil^22^, monocyte/macrophage^23^, and complement fixation^24^ which seems to be similar to diabetes condition^47^. Furthermore, a cohort study of Thai adult patients with thalassaemia reported higher prevalence of infection in transfusion dependent thalassaemia (TDT) than non-transfusion dependent thalassaemia (NTDT)^34^. Our results correlated with a previous study reporting no significant difference of IFN-γ production between splenectomized and non-splenectomized β-thalassaemia patients with TDT^19^. Furthermore, splenectomy had benefit probably in some TDT patients, but advanced age and splenectomy were the significant clinical risk factors for most of the disease-related complications in both TDT and NTDT groups^34,48^.

Further experiments revealed that activation of IFN-γ production via T cell receptor (TCR) signal by using anti-CD3 and anti-CD28 antibodies stimulated on whole blood from β-thalassaemia patients is higher than healthy controls. This data is consistent with previous reports that β-thalassaemia patients have increased expression of CD3 marker on T cell surface than healthy controls^19^, and also T cell activation is not defective^42^, although dysfunction of T and B cells have been reported in conditions with high levels of ferritin^19,49^. Moreover, concentrations of anti-Bp serum IgG from β-thalassaemia patients was comparable to healthy controls, while total antibody levels in serum of β-thalassaemia patients was increasing^50^. Therefore, based on our results, there is no significant general impairment of immune responses through TCR and IgG antibody production against Bp from β-thalassaemia patients in comparison to healthy controls.

The hemolytic consequences of β-thalassaemia lead to increasing levels of heme in blood circulation^25^ which causes cell death by inducing free radical oxidative species (ROS)^51–53^ and elicit inflammatory injuries^51,54^. HO-1 is upregulated to catalyze heme into iron, carbon monoxide and bilirubin^55^. Expression of HO-1 is not only for heme catalysis but can also adversely affect immune function. Studies using mouse derived macrophages show increasing level of *HO-1* mRNA expression after infected with Bp, and leading to increase serum IL-6, TNF-α and MCP-1, but IFN-γ production was decreased^29^. Here we found increased *HO-1* mRNA expression in whole blood from β-thalassaemia patients, which increased further in the presence of Bp in vitro. The presence of increased baseline levels of *HO-1* in β-thalassaemia can be explained by the presence of high levels of hemin (> 50 μM) in serum from β-thalassaemia patients, but undetectable in non-thalassaemia individuals^25^. Hemin and products of hemin degradation by HO-1 can have multiple effects on cellular immune functions. CD4 T cell and monocyte coculture in presence of hemin triggers a polarization of CD4 T cells to a regulatory T cell (Treg) subset^56^. A study of carbon monoxide releasing molecule (CORM-2) treated C57BL/6 mice infected with Bp revealed increasing burden of bacteria in lung and increased bacterial load in macrophages^29^. Carbon monoxide is also able to suppress human CD4 T cell proliferation and IL-2 secretion^57^. In addition, in a mouse model HO-1 mediated iron released from catabolism of heme leads to cell damage in thalassaemic erythroblasts^58^. Taken together, all studies lead to our hypothesis that hemin induced HO-1 reaction may explain immune response impairment and increasing of susceptibility to infection.

To further test the effects of HO-1 per se on immune function, we investigated the effect of chemical induced HO-1 activation or inhibition before stimulation of leucocytes from healthy individuals with *B. pseudomallei*. Treatment of normal human PBMCs with HO-1 activators, hemin and CoPP, caused reduction of IFN-γ and IL-10 after stimulation with *B. pseudomallei*. In contrast inhibition of HO-1 by using SnPP increased IFN-γ production with no effect on IL-10. This is in comparison with studies in mice showing that HO-1 activation by CoPP impaired dendritic cell maturation, CD4^+^ and CD8^+^ T-cell proliferation and IFN-γ production but increased IL-10 in response to LPS in vitro^59^. HO-1 can also inhibit IFN-γ, decrease immune cell numbers and also induce T cell apoptosis of mice^60^.

To investigate the consequences of these changes to anti-bacterial immunity, we measured cell mediated responses induced by live pathogenic bacteria (namely *B. pseudomallei*, *E. coli*, *S. typhimurium* and *S. aureus*) which cause serious disease in patients with thalassaemia in Thailand^2^. The HO-1 activators, hemin and CoPP impaired bacterial killing activity of human whole blood and purified monocytes from healthy controls, which correlated with low production of IFN-γ, in all bacteria tested. In contrast, the HO-1 inhibitor, SnPP enhanced bacterial killing and resulted in higher levels of IFN-γ compared with medium controls. Interestingly whole blood from β-thalassaemia patients impaired bactericidal activities upon exogenous HO-1 induced by hemin or CoPP, similar to healthy controls. However HO-1 inhibition by SnPP did not enhance bactericidal activity suggesting that high levels of endogenous HO-1 might not be completely inhibited under this condition or there might be other mechanisms to impair bactericidal activity in β-thalassaemia. This issue still needs further investigation. Apart from our observation, there are more reported bacterial clearance mechanisms that HO-1 can be modulated by hemin and strongly inhibited phagocytic activity and migration of human monocyte and neutrophil, which is not correlated with heme–iron catabolism^26^. HO-1 expression can promote *M. tuberculosis* growth within primary human macrophage cells^30^, and also reduce bacterial killing in mice infected with *B. pseudomallei*^29^. Induction of HO-1 expression by CoPP combined with LPS can inhibit T cell responses and reduce IFN-γ production by impairing DC maturation and expression of MHC-II, CD40, CD80, and CD86 in mice^59^.

In conclusion, our study demonstrates defective cytokine production of β-thalassaemia patients in response to a diverse range of stimuli derived from pathogenic bacteria. We believe that HO-1, as well as hemin, is primarily responsible for these effects on production of both IFN-γ and IL-10, and also impair the bactericidal activity of human leucocytes against pathogens known to infect individuals with β-thalassaemia. Modulation of the heme/HO-1 pathway may hold promise for future host directed therapies. Protection of macrophage cell lines and human macrophages, by quinine can prevent damaging effects of increasingly endogenous heme following sepsis with gram negative bacteria and restore macrophage phagocytosis in vivo^26^. In mouse models of β-thalassaemia, inhibition of HO-1 by SnPP can decrease heme catabolism and reduce iron release including improved erythropoiesis^61^. Blocking of HO-1 expression may be an alternative therapy for septic β-thalassaemia patients to reduce heme catabolism and enhance proinflammatory cytokines for improved bacterial killing.

##  Methods

### Subjects

This study was approved by Human Ethics Committee at Nakhon Phanom Hospital, IEC-NKP1-No.19/2558. Informed consent was obtained from all participants and in compliance with the Declaration of Helsinki. Healthy donors were defined by blood bank guidelines and had no signs of infection at the time of blood collection. β-thalassaemia patients were previously diagnosed by clinicians, and blood samples were collected before receiving blood transfusion. All donors with mean corpuscular volume (MCV) < 80 fL and dichlorophenol indophenol precipitation (DCIP) positive were confirmed by Hb typing and classified as HbE carriers^62^. Diabetes mellitus (DM) individuals were defined as type 2 DM by clinicians and had fasting blood sugar more than 126 mg/dl^63^ at the time of blood collection.

### Bacteria preparation

Live Bp strain K96243, *E. coli* ATCC25922, *Salmonella enterica* serovar Typhimurium ATCC13311, *S. aureus* ATCC25923 were grown in mid log phase and the number of bacteria was confirmed by colony count. Live Bp were killed by 2% paraformaldehyde (PFA) and kept frozen at − 80 °C until use.

### Cell culture

Whole blood samples were collected as previously published protocol^4^ and incubated with medium alone, 10 μg/ml *E. coli* lipopolysaccharide (LPS; Sigma), 5.4 × 10^6^ CFUs PFA fixed Bp (ratio at 30:1), or 10 μg/ml BPSL0280 (FlgK) protein. After 3 h at 37 °C, cell pellets were collected for HO-1 expression analysis by real-time PCR and supernatants were collected at 48 h. In some experiment, adjusted whole blood samples were cultured at 37 °C in the presence of 3 μg/ml anti-CD3 (eBioscience) and 3 μg/ml anti-CD28 (eBioscience), or in the presence of 3 μg/ml isotype antibody (eBioscience). IFN-γ and IL-10 levels in supernatant were detected by ELISA kits (BD Biosciences) following the manufacturer’s instructions.

In other experiments, PBMCs or CD14 positive cells were isolated from whole blood samples^4^ and plated at 2.5 × 10^5^ cells/ml, then pretreated for 3 h at 37 °C with vehicle control (0.1 M NaOH; Sigma), a substrate of HO-1 (hemin; Sigma), an HO-1 inducer (cobalt protoporphyrin IX (CoPP); Enzo Life Sciences), or an HO-1 inhibitor (tin protoporphyrin IX (SnPP); Enzo Life Sciences). Sequentially, 7.5 × 10^6^ CFUs of PFA fixed Bp (ratio at 30:1) were cultured with pretreated PBMCs or monocytes for 48 h at 37 °C. After 3 h of pre-treatment at 37 °C, cell pellets were collected for HO-1 expression analysis by real-time PCR and after 48 h supernatants were collected for cytokine quantification by ELISA.

### Plasma IgG antibody detection

PFA fixed Bp at 10^6^ CFUs per well were coated onto 96 wells ELISA plates overnight before adding 1:300 diluted heparinized plasma in duplicate and following our published protocol^64–66^.

### Measurement of *HO-1* mRNA expression by quantitative real-time polymerase chain reaction

Total RNA from whole blood was isolated using Tempus Spin RNA Isolation Kit (Tempus), while total RNA from cell culture pellets was isolated using RNeasy Mini Kit (Qiagen) following the manufacturer’s instructions. Reverse transcription of 1 μg total RNA was done by using ImProm-II Reverse Transcription System (Promega). Quantitative real-time PCR was performed by using Rotor gene 3000, with AccuPower 2X GreenstarTM qPCR Master Mix (Bioneer). *GADPH* was used as internal reference gene^67^. *HO-1* amplicons were detected by using forward primer 5′-GCAGAGAATGCTGAGTTCATG-3′ and reverse primer 5′-CACATCTATGTGGCCCTGGAGGAGG-3^68^. All assays were performed in duplicate and the results were shown as cycle threshold (Ct).

### Bacterial clearance assay

After pretreatment for 3 h with vehicle control, 25 μM hemin, 50 μM CoPP, or 10 μM SnPP, whole blood or monocytes were infected with 10^5^ CFUs and 5 × 10^5^ CFUs, respectively, of live Bp, *E. coli*, *S. enterica* serovar Typhimurium or *S. aureus* for 1, 3 and 6 h at 37 °C. At each time point, infected cells were lysed with 1% Triton X-100 (Biotech), then total numbers of viable bacteria were counted by colony count on LB agar plate. Data was analyzed as % Bacterial killing = ((inoculum bacteria – remaining bacteria)/inoculum bacteria) × 100.

### Data analysis

All statistical analysis was done by using Graphpad Prism version 6 (Graphpad software). One-way ANOVA was applied for independent sample sets, while two-way ANOVA was applied for matched sample sets. Wilcoxon matched-pair’s signed rank test was applied for comparison of non-normally distribution data, whereas Mann Whitney test was applied for comparison of independent patients. Statistically significant differences were considered at *P* < 0.05.

## Supplementary information

Supplementary file.

## References

[1] Weatherall DJ (2010). The inherited diseases of hemoglobin are an emerging global health burden. Blood.

[2] Teawtrakul N, Jetsrisuparb A, Sirijerachai C, Chansung K, Wanitpongpun C (2015). Severe bacterial infections in patients with non-transfusion-dependent thalassemia: Prevalence and clinical risk factors. Int J Infect Dis.

[3] Suputtamongkol Y (1999). Risk factors for melioidosis and bacteremic melioidosis. Clin Infect Dis.

[4] Tippayawat P (2009). Phenotypic and functional characterization of human memory T cell responses to *Burkholderia pseudomallei*. PLoS Negl Trop Dis.

[5] Patel N (2011). Development of vaccines against *Burkholderia pseudomallei*. Front Microbiol.

[6] Jenjaroen K (2015). T-cell responses are associated with survival in acute melioidosis patients. PLoS Negl Trop Dis.

[7] Haque A (2006). Role of T cells in innate and adaptive immunity against murine *Burkholderia pseudomallei* infection. J Infect Dis.

[8] Wiersinga WJ (2007). High-throughput mRNA profiling characterizes the expression of inflammatory molecules in sepsis caused by *Burkholderia pseudomallei*. Infect Immun.

[9] Mulye M (2014). Delineating the importance of serum opsonins and the bacterial capsule in affecting the uptake and killing of *Burkholderia pseudomallei* by murine neutrophils and macrophages. PLoS Negl Trop Dis.

[10] Hatcher CL, Muruato LA, Torres AG (2015). Recent advances in *Burkholderia mallei* and *B. pseudomallei* research. Curr Trop Med Rep.

[11] Santanirand P, Harley VS, Dance DA, Drasar BS, Bancroft GJ (1999). Obligatory role of gamma interferon for host survival in a murine model of infection with *Burkholderia pseudomallei*. Infect Immun.

[12] Hodgson KA, Govan BL, Walduck AK, Ketheesan N, Morris JL (2013). Impaired early cytokine responses at the site of infection in a murine model of type 2 diabetes and melioidosis comorbidity. Infect Immun.

[13] Chaudhry H (2013). Role of cytokines as a double-edged sword in sepsis. In Vivo.

[14] Romero CR (2010). The role of interferon-gamma in the pathogenesis of acute intra-abdominal sepsis. J Leukoc Biol.

[15] Kessler B (2017). Interleukin 10 inhibits pro-inflammatory cytokine responses and killing of *Burkholderia pseudomallei*. Sci Rep.

[16] Kaewarpai T (2019). Longitudinal profiling of plasma cytokines in melioidosis and their association with mortality: A prospective cohort study. Clin Microbiol Infect.

[17] Vento S, Cainelli F, Cesario F (2006). Infections and thalassaemia. Lancet Infect Dis.

[18] Ricerca BM, Di Girolamo A, Rund D (2009). Infections in thalassemia and hemoglobinopathies: Focus on therapy-related complications. Mediterr J Hematol Infect Dis.

[19] Gharagozloo M, Karimi M, Amirghofran Z (2009). Double-faced cell-mediated immunity in beta-thalassemia major: Stimulated phenotype versus suppressed activity. Ann Hematol.

[20] Speer CP, Gahr M, Schuff-Werner P, Schroter W (1990). Immunologic evaluation of children with homozygous beta-thalassemia treated with desferrioxamine. Acta Haematol.

[21] Ezer U, Gulderen F, Culha VK, Akgul N, Gurbuz O (2002). Immunological status of thalassemia syndrome. Pediatr Hematol Oncol.

[22] Matzner Y (1993). Impaired neutrophil chemotaxis in patients with thalassaemia major. Br J Haematol.

[23] Sternbach MS, Tsoukas C, Paquin M, Lajeunesse N, Strawczynski H (1987). Monocyte–macrophage (M–M) functions in asymptomatic hemophiliacs and supertransfused thalassemics. Clin Invest Med.

[24] Sinniah D, Yadav M (1981). Elevated IgG and decreased complement component C3 and factor B in B-thalassaemia major. Acta Paediatr Scand.

[25] Phumala N (2003). Hemin: A possible cause of oxidative stress in blood circulation of beta-thalassemia/hemoglobin E disease. Free Radic Res.

[26] Martins R (2016). Heme drives hemolysis-induced susceptibility to infection via disruption of phagocyte functions. Nat Immunol.

[27] Chung SW, Hall SR, Perrella MA (2009). Role of haem oxygenase-1 in microbial host defence. Cell Microbiol.

[28] Gozzelino R, Jeney V, Soares MP (2010). Mechanisms of cell protection by heme oxygenase-1. Annu Rev Pharmacol Toxicol.

[29] Stolt C, Schmidt IH, Sayfart Y, Steinmetz I, Bast A (2016). Heme oxygenase-1 and carbon monoxide promote *Burkholderia pseudomallei* infection. J Immunol.

[30] Scharn CR (2016). Heme oxygenase-1 regulates inflammation and mycobacterial survival in human macrophages during *Mycobacterium tuberculosis* infection. J Immunol.

[31] Abdalla MY, Ahmad IM, Switzer B, Britigan BE (2015). Induction of heme oxygenase-1 contributes to survival of *Mycobacterium abscessus* in human macrophages-like THP-1 cells. Redox Biol.

[32] Faure E (2001). Bacterial lipopolysaccharide and IFN-gamma induce Toll-like receptor 2 and Toll-like receptor 4 expression in human endothelial cells: role of NF-kappa B activation. J Immunol.

[33] Cappellini MD, Cohen A, Porter J, Taher A, Viprakasit V (2014). Guidelines for the Management of Transfusion Dependent Thalassaemia (TDT).

[34] Chuncharunee S, Teawtrakul N, Siritanaratkul N, Chueamuangphan N (2019). Review of disease-related complications and management in adult patients with thalassemia: A multi-center study in Thailand. PLoS ONE.

[35] Itoh T (2001). Hemin (Fe(3+))- and heme (Fe(2+))-smectite conjugates as a model of hemoprotein based on spectrophotometry. Bioconjug Chem.

[36] Martin PL (2019). Heme oxygenase-1 induction by hemin prevents oxidative stress-induced acute cholestasis in the rat. Clin Sci (Lond).

[37] Campbell NK (2018). Naturally derived heme-oxygenase 1 inducers attenuate inflammatory responses in human dendritic cells and T cells: Relevance for psoriasis treatment. Sci Rep.

[38] Godai K, Kanmura Y (2018). Heme oxygenase-1 inducer and carbon monoxide-releasing molecule enhance the effects of gabapentinoids by modulating glial activation during neuropathic pain in mice. Pain Rep.

[39] Danjou F, Anni F, Galanello R (2011). Beta-thalassemia: From genotype to phenotype. Haematologica.

[40] Taher AT, Weatherall DJ, Cappellini MD (2018). Thalassaemia. Lancet.

[41] Singh DK, Winocour P, Farrington K (2009). Erythropoietic stress and anemia in diabetes mellitus. Nat Rev Endocrinol.

[42] Pattanapanyasat K (2000). Lymphocyte subsets and specific T-cell immune response in thalassemia. Cytometry.

[43] Dunachie SJ (2017). Infection with *Burkholderia pseudomallei*—immune correlates of survival in acute melioidosis. Sci Rep.

[44] Wanachiwanawin W (1999). Serum levels of tumor necrosis factor-alpha, interleukin-1, and interferon-gamma in beta(o)-thalassemia/HbE and their clinical significance. J Interferon Cytokine Res.

[45] Fabbri E (2015). Aging and the burden of multimorbidity: Associations with inflammatory and anabolic hormonal biomarkers. J Gerontol A Biol Sci Med Sci.

[46] Lu YC, Yeh WC, Ohashi PS (2008). LPS/TLR4 signal transduction pathway. Cytokine.

[47] Wiersinga WJ (2018). Melioidosis. Nat Rev Dis Primers.

[48] Tantiworawit A (2018). The pros and cons of splenectomy in transfusion dependent thalassemia patient. Blood.

[49] Ghaffari J, Vahidshahi K, Kosaryan M, Soltantooyeh Z, Mohamadi M (2011). Humoral immune system state in β thalassemia major. Med Glas (Zenica).

[50] Akbar AN, Giardina PJ, Hilgartner MW, Grady RW (1985). Immunological abnormalities in thalassaemia major. I. A transfusion-related increase in circulating cytoplasmic immunoglobulin-positive cells. Clin Exp Immunol.

[51] Dutra FF, Bozza MT (2014). Heme on innate immunity and inflammation. Front Pharmacol.

[52] Fortes GB (2012). Heme induces programmed necrosis on macrophages through autocrine TNF and ROS production. Blood.

[53] Kitatsuji C (2016). Protein oxidation mediated by heme-induced active site conversion specific for heme-regulated transcription factor, iron response regulator. Sci Rep.

[54] Lin S (2012). Heme activates TLR4-mediated inflammatory injury via MyD88/TRIF signaling pathway in intracerebral hemorrhage. J Neuroinflammation.

[55] Ryter SW, Alam J, Choi AM (2006). Heme oxygenase-1/carbon monoxide: From basic science to therapeutic applications. Physiol Rev.

[56] Zhong H, Bao W, Friedman D, Yazdanbakhsh K (2014). Hemin controls T cell polarization in sickle cell alloimmunization. J Immunol.

[57] Pae HO (2004). Carbon monoxide produced by heme oxygenase-1 suppresses T cell proliferation via inhibition of IL-2 production. J Immunol.

[58] Santos DG, Mikhael M, Rivella S (2015). Heme oxygssenase 1 plays a role in the pathophysiology of beta-thalassemia. Blood.

[59] Zhao Y (2018). Upregulation of heme oxygenase-1 endues immature dendritic cells with more potent and durable immunoregulatory properties and promotes engraftment in a stringent mouse cardiac allotransplant model. Front Immunol.

[60] Yoon SJ, Kim SJ, Lee SM (2017). Overexpression of HO-1 contributes to sepsis-induced immunosuppression by modulating the Th1/Th2 balance and regulatory t-cell function. J Infect Dis.

[61] Garcia-Santos D (2018). Inhibition of heme oxygenase ameliorates anemia and reduces iron overload in a beta-thalassemia mouse model. Blood.

[62] Prayongratana K, Polprasert C, Raungrongmorakot K, Tatone K, Santiwatanakul S (2008). Low cost combination of DCIP and MCV was better than that of DCIP and OF in the screening for hemoglobin E. J Med Assoc Thai.

[63] Aekplakorn W (2011). Prevalence and management of diabetes and metabolic risk factors in Thai adults: the Thai National Health Examination Survey IV, 2009. Diabetes Care.

[64] Gori A (2016). Flexible vs rigid epitope conformations for diagnostic- and vaccine-oriented applications: novel insights from the *Burkholderia pseudomallei* BPSL2765 Pal3 Epitope. ACS Infect Dis.

[65] Gourlay LJ (2015). From crystal structure to in silico epitope discovery in the *Burkholderia pseudomallei* flagellar hook-associated protein FlgK. FEBS J.

[66] Nithichanon A (2015). Sequence- and structure-based immunoreactive epitope discovery for *Burkholderia pseudomallei* flagellin. PLoS Negl Trop Dis.

[67] Kewcharoenwong C (2013). Glibenclamide reduces pro-inflammatory cytokine production by neutrophils of diabetes patients in response to bacterial infection. Sci Rep.

[68] Rushworth SA, Chen XL, Mackman N, Ogborne RM, O'Connell MA (2005). Lipopolysaccharide-induced heme oxygenase-1 expression in human monocytic cells is mediated via Nrf2 and protein kinase C. J Immunol.

