# Supplementary material for: Immune responses in beta-thalassaemia: heme oxygenase 1 reduces cytokine production and bactericidal activity of human leucocytes
Source: Sci Rep. 2020 Jun 24;10:10297. doi: 10.1038/s41598-020-67346-2 (PMC7314746; doi:10.1038/s41598-020-67346-2)
Supplement: Supplementary file 1 — Supplementary file. [file 41598_2020_67346_MOESM1_ESM.pdf]

Supplementary data

Immune Responses in Beta-thalassaemia: Heme Oxygenase 1 Reduces Cytokine Production and Bactericidal Activity of Human Leucocytes

Arnone Nithichanon[1,#], Inthira Tussakhon[1,#], Waraporn Samer[1], Chidchamai Kewcharoenwong[1], Manabu Ato[2], Gregory J. Bancroft[3], Ganjana Lertmemongkolchai[1,*].

Affiliations

[1]The Centre for Research and Development of Medical Diagnostic Laboratories, Faculty of Associated Medical Sciences, Khon Kaen University, Khon Kaen 40002, Thailand

[2]Department of Immunology, National Institute of Infectious Diseases, Tokyo 162-8640, Japan

[3]Department of Infection Biology, London School of Hygiene and Tropical Medicine, London WC1E 7HT, UK

# equal contribution

***Correspondence**

Ganjana Lertmemongkolchai

Tel: +66 4320 3825; Fax: +66 4320 3826; E-mail: ganja_le@kku.ac.th

Table S1: Comparison of β-thalassaemia/HbE patients in this study by phenotypic classification

**β-thalassaemia/HbE (n = 16)**	**TDT (n = 7)**	**NTDT (n = 9)**
Splenectomy, n (%)		
No	3 (43%)	7 (88%)
Yes	4 (57%)	2 (22%)
Ferritin level (ng/ml)[#]	1713* (1485-3024)	784 (370-1948)

[#]median with interquartile range

*$p < 0.05$, Mann-Whitney test

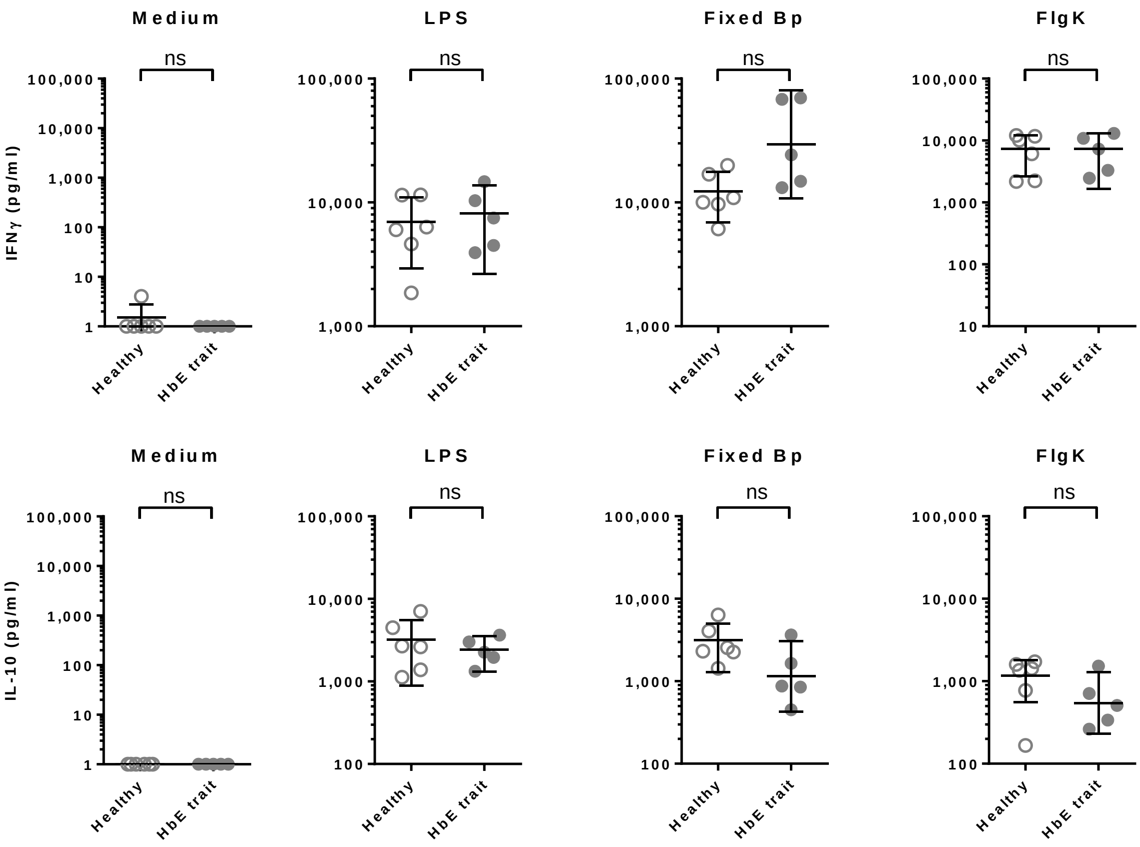

Figure S1: Comparisons of IFN-γ and IL-10 level from whole blood samples of healthy individuals with or without thalassaemia carrier phenotype upon stimulation. Whole blood samples (adjusted the number of lymphocyte plus monocyte at 1.8 x 10^5 cells) from healthy controls (○, n = 6) and healthy with HbE thalassaemia carrier phenotype; HbE trait (●, n = 5) were cultured with medium alone, 10 μg/ml of LPS, 5.4 x 10^6 CFUs PFA fixed Bp (ratio at 30:1) or 10 μg/ml FlgK protein for 48 hours. IFN-γ and IL-10 production upon stimulation in supernatant were measured by ELISA, and the results were shown as scattered dot plot and line at mean with 95% confidence interval. Statistical significance was analyzed by using student's t test; * $P < 0.05$, ** $P < 0.01$, *** $P < 0.001$, and ns, non-significant.

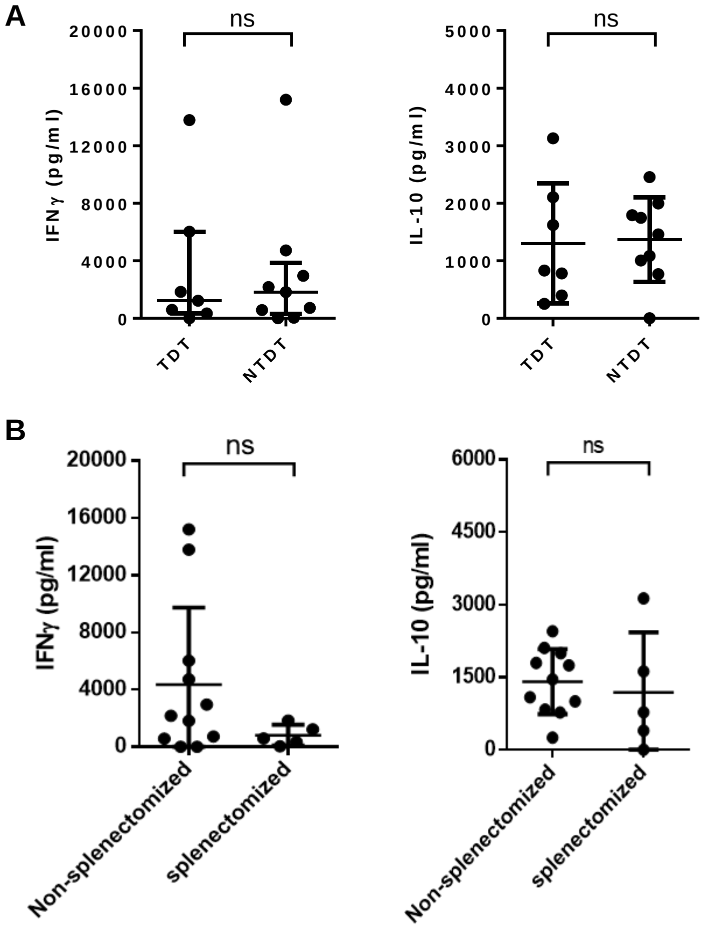

Figure S2: Comparisons of IFN-γ and IL-10 level from whole blood samples of β-thalassaemia/HbE patients with different clinical outcomes upon stimulation. Whole blood samples (adjusted the number of lymphocyte plus monocyte at 1.8 x 10^5 cells) from β-thalassaemia/HbE patients (n = 16) were cultured with 5.4 x 10^6 CFUs PFA fixed Bp (ratio at 30:1) for 48 hours. IFNγ and IL-10 production upon stimulation in supernatant were measured by ELISA. The results compared between transfusion dependent thalassaemia (TDT) versus non-transfusion dependent thalassaemia (NTDT) **(A)** or non-splenectonized thalassaemia versus splenectonized thalassaemia **(B)** were shown as scattered dot plot with line at median with 95% confidence interval. Statistical significance was analyzed by using student's t test; ns, non-significant.

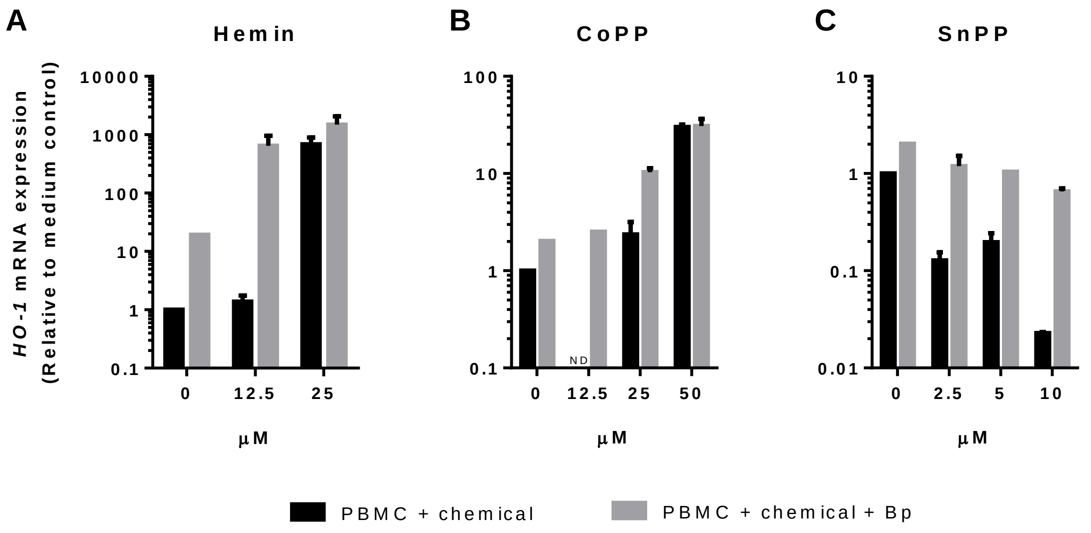

Figure S3: *HO-1* mRNA expression after pretreated human PBMCs with Hemin, CoPP and SnPP *in vitro*. Isolated human PBMCs were pretreated with hemin **(A)**, CoPP **(B),** or SnPP **(C)** for 3 hours at various concentrations before culture with PFA fixed Bp for another 3 hours. *HO-1* mRNA expression was analyzed by real-time PCR using *GADPH* as internal reference gene, ΔCt between *HO-1* and *GADPH*, then ΔΔCt comparing to vehicle control (0 μM) were calculated before calculated for *HO-1* mRNA expression by $2^{-\Delta\Delta Ct}$. Results were shown as bar graph with error bar from indicated HO-1 mRNA expression in duplicate.

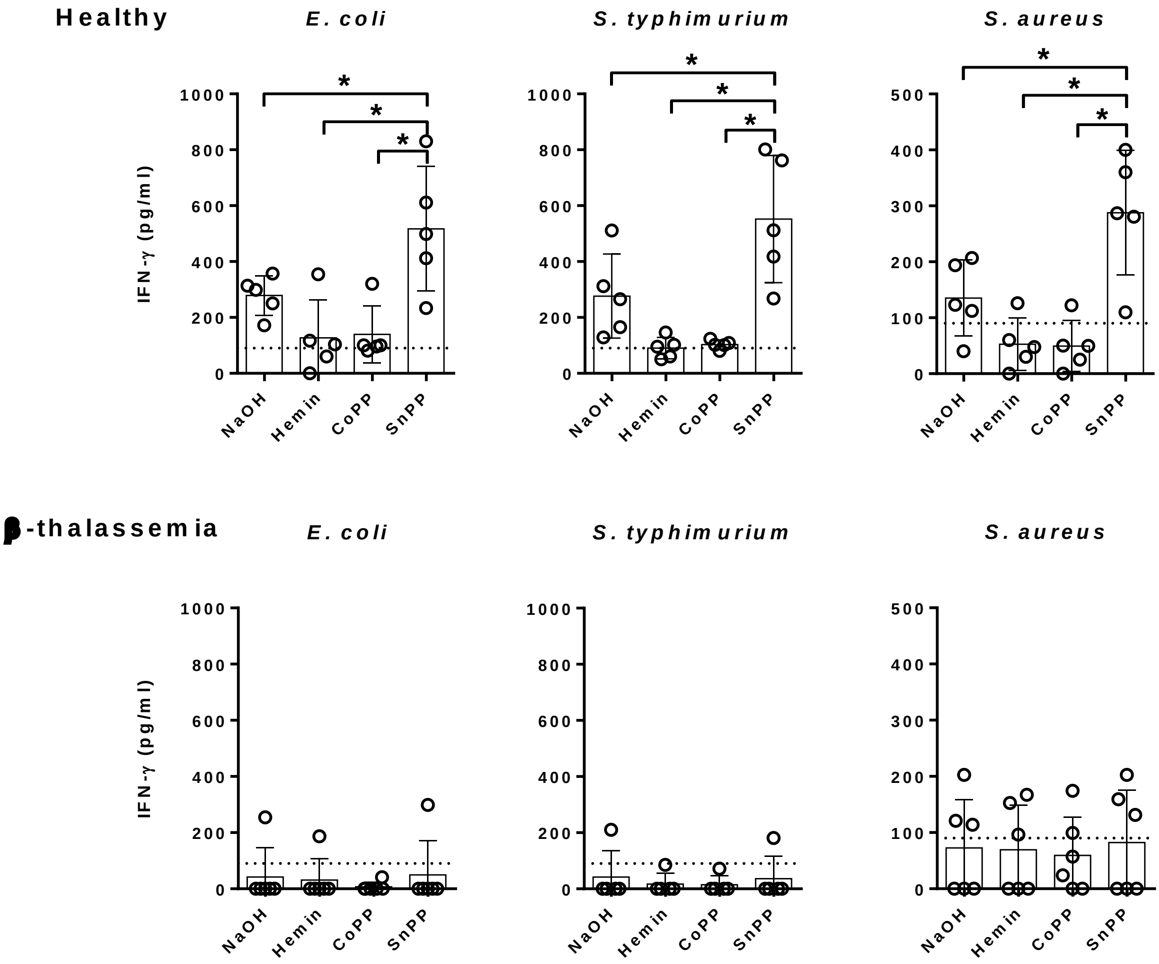

Figure S4: Effect of HO-1 expression on IFN-γ production in human whole blood. Whole blood from healthy donors (n = 5) or β-thalassemia (β-thal; n = 6) were pre-treated 3 hours with medium control (NaOH), hemin at 25 μM, CoPP at 50 μM or SnPP at 10 μM before infected with 10^5 CFUs of *E. coli*, *S. typhimurium* or *S. aureus*. Supernatants from 24 hours infection culture were collected for IFN-γ quantification. Dash-lines represent the lower limit of detection by ELISA. Statistical significance was analyzed by using one-way ANOVA with Tukey's multiple comparisons test; * $P < 0.05$.
